# Recent Innovations in Eating Disorder Prevention and Early Intervention

**DOI:** 10.1007/s11920-025-01657-x

**Published:** 2025-12-26

**Authors:** Kennedi Burton, Jennifer Finkelstein, Tiffany A. Brown

**Affiliations:** Department of Psychological Sciences, Auburn University, 226 Thach Hall, Auburn, AL 63849 USA

**Keywords:** Eating disorders, Disordered eating, Prevention, Early intervention, Review

## Abstract

**Purpose of Review:**

The purpose of this review is to synthesize recent advancements over the last four years in eating disorder prevention and early intervention research.

**Recent Findings:**

Our comprehensive review identified over 140 prevention and early intervention articles published from 2021 to 2024, with themes focused on specific intervention strategies or targets (e.g., dissonance, mindfulness, weight stigma), targeted populations (e.g., adolescents/school-based programs, athletes), digital-based interventions, system-level interventions, and early interventions.

**Summary:**

The eating disorder prevention and early intervention literature has expanded over the last several years. Based on the current state of the evidence, we outline future directions to help inform the field and continue movement towards accessible and inclusive eating disorder prevention.

## Introduction

Eating disorders are multifaceted, complex mental health conditions that wreak havoc on one’s psychological and physical health and inflict substantial economic burden on individuals and healthcare systems [1–3]. While evidence-based treatments across eating disorders exist, numerous barriers to access (e.g., treatment cost, provider availability) prevent many who need help from receiving services [4]. Even among those who do receive empirically-supported care, remission rates remain unfortunately low [5]. One solution is to intervene before the individual fully develops an eating disorder through prevention and early intervention programs. Prevention efforts strive to reduce risk factors and promote protective factors in individuals without full threshold eating disorders. Notably, most studies in this literature have been unable to assess whether these prevention programs ultimately reduce eating disorder onset, given the extensive time and resources required. According to the Institute of Medicine, prevention programs can be further parsed into *universal* (i.e., programs catered to all populations), *selective* (i.e., programs designed for at-risk sub-populations), and *indicated* programs (i.e., programs for individuals with eating disorder symptoms). Early intervention programs are designed for individuals with recent eating disorder onset to intervene before disorder progression/chronicity. While prior reviews in the last several years have synthesized various aspects of eating disorder prevention [6, 7], the present article sought to synthesize the prevention and early intervention efforts from 2021 to 2024, building upon a prior rapid review through 2021 [7].

## Methods

Although the present study was not a formal systematic review, a comprehensive literature search was conducted in PsycInfo, using the following terms: eating disorder*, prevention*, early intervention* for data papers written in English and published in peer-reviewed journals from 2021 to 2024. The authors also reviewed reference sections of relevant articles to ensure a thorough review process. After duplicates and irrelevant articles to the focus of the review were removed, over 140 eating disorder prevention or early intervention articles were identified, indicating a thriving literature. Due to the scope and space limitations, 47 articles were not included in the text of this review. Examples of excluded articles include additional adaptations of dissonance-based programs and school-based programs designed to prevent both eating disorders and weight gain. Remaining articles were categorized by the author team into several notable topics focused on specific intervention strategies or targets (e.g., dissonance, mindfulness, weight stigma), targeted populations (e.g., adolescents/school-based programs, athletes), digital interventions, system-level interventions, and early intervention (see Table 1). Of note, many articles intersected with multiple categories (e.g., digital interventions in youth, dissonance-based programs on reducing weight stigma), and authors organized articles into groupings that captured the primary focus of the article.

**Table 1:** Overview of eating disorder prevention and early intervention programs from 2021 to 2024

Program Type	Sample of Interventions	Populations Targeted	Approach/ Components	Delivery Format	Level of Evidence (Number of Studies per type of Trial)
Specific Intervention Strategies or Targets					
Dissonance-Based	Body Project (and variants)	• Adolescents, college students (across globe)	• Cognitive dissonance, peer-led	In-person group, virtual group	• Implementation RCT (3) • RCT w/ time/attention ctrl (3) • RCT w/ active ctrl (3) • RCTs w/ WL/assessment ctrl (7) • Non-randomized vs.TAU ctrl (1) • Open trial (7)
Mindfulness-Based	Eat Breathe Thrive (EBT), Self-Compassion	• General samples, athletes, cancer patients	• Yoga, intuitive eating, self-compassion exercises	In-person group, virtual, brief writing	• RCT w/ WL/assessment ctrl (2) • RCT w/ time-match ctrl (3) • Quasi-experimental (3) • Open/uncontrolled trial (2)
Weight Stigma-Focused	Body Advocacy Movement, CBT, Compassion	• Higher-weight individuals, general samples	• Narratives, CBT, dissonance, empathy-building	In-person group sessions, online	• RCT w/ TAU control (1) • RCT w/ education brochure (1) • RCT w/ waitlist control (1) • Quasi-experimental w/ active ctrl (1) • Open Trial (1)
**Specific Populations**					
Youth/School-Based	SoMe, CBCC, PRIORITY, Goodform	• School-aged youth, parents, boys, adolescents with Type-I diabetes	• Social media literacy, psychoeducation	Classroom-based, parent-focused	• RCTs with active ctrl (1) • RCT w/ TAU (3) • RCT w/ WL/assessment ctrl (2) • Open trial (3)
Athlete-Focused	Female Athlete Body Project, MOPED-A	• Athletes, dancers, exercisers	• Peer-led, nutrition, body ideals, motivation	In-person group, Online self-help	• RCT w/ WL/assessment ctrl (2) • Open Trial (3)
**Digital-Based**,** Systems-Level**,** and Early Intervention**					
Digital-Based Interventions	Chatbots, Apps, SSI, CBT-Online	• At-risk individuals, general samples	• Self-help, CBT, single-session, AI chatbots	Mobile/web-based	• RCT w/ active ctrl (1) • RCT w/ TAU ctrl (1) • RCT w/ WL/assessment ctrl (6) • Open trials (4)
Systems-Level	CBCC, MCM, legislature	• Medical centers, community health and advocates	• Primary care training, public policy, research	Clinic-based, community, legal	• Qualitative/process evaluation (3) • Policy evaluation (1) • Cost-effectiveness analysis (1) • Implementation hybrid (1)
Early Intervention	FREED, emerge-ED, EDIFY	• newly diagnosed, family members, clinicians, parents	• Group therapy, intervention model, parent groups	In-person, online, hospital and clinic-based,	• Quasi-experimental implementation vs. TAU (2) • Implementation trial (2) • Open Trial (2) • Qualitative (1) • Proposed Framework (1)

*Ctrl* control; *RCT* randomized controlled trial; *TAU* treatment as usual; *w/* with; *WL* waitlist

### Specific Intervention Strategies or Targets

#### Dissonance-Based Approaches

Dissonance-based eating disorder prevention programs (i.e., the Body Project), are an extensively researched and a front-line prevention program, with evidence demonstrating reductions in eating disorder risk factors and onset across multiple studies, with effects persisting through 3-year follow-up [8]. These group, peer-led programs are based on cognitive dissonance theory and aim to reduce internalization of the appearance ideal, thus lessening risk factors associated with the development of eating disorders [8]. The Body Project is also one of the few programs that actually prevents future eating disorder onset, with a meta-analytic review indicating a 54–77% reduction in eating disorder onset compared to alternative controls [6]. These effects may be strongest for preventing purging disorder (OR 0.35) and bulimia nervosa (OR 0.47), with non-significant effects for subthreshold/threshold anorexia nervosa (OR 0.56) and binge eating disorder (OR 0.70), compared to expressive writing controls through 4-year follow-up [9]. The Body Project can also be delivered virtually, with a recent RCT indicating that virtual delivery yielded significant moderate-to-large reductions in eating disorder risk factors (*d* = 0.56–0.94.56.94) at 1-month follow-up compared to waitlist control, which was 49% larger than effects in previous in-person groups [10].

In the last four years, over 40 articles have been published on dissonance-based programs, with a substantial focus on adapting and implementing programming to better serve diverse populations across the globe including: Brazilian men [11, 12], Brazilian sexual minority men [13], adult women in Saudi Arabia [14, 15], Switzerland [16], and China [17], Mexican university students [18], adolescent girls in Argentina [19], Australia [20], India [21] and the UK [22], Orthodox Jewish girls [23], women with type 1 diabetes [24, 25], and adult women over the age of 25 [26]. These adaptations typically retain the core components/structure of the Body Project with relatively superficial adjustments (e.g., wording and slight content changes) to better accommodate diverse demographics. Consistent with this trend, the EVERYbody Project adapted the Body Project materials to be gender-inclusive and diversity-focused and has been tested in both middle-schoolers and college students [27–29]. Results from a randomized controlled trial (RCT) indicate that the EVERYbody Project demonstrated significant improvements in appearance internalization (*d* = 0.42), body dissatisfaction (*d* = 0.18), and negative affect (*d* = 0.65) compared to video control through 3-month follow-up in college students [28]. Another adaptation is the Diabetes Body Project, which adds sessions covering diabetes-specific content (e.g., benefits of diabetes self-care, insulin use). Results from an uncontrolled pilot study reported significant, large reductions in Type-1 diabetes eating disorder symptoms (*d* = 1.45), body dissatisfaction (*d* = 1.48), and appearance ideals and internalizations (*d* = 1.18) through 6-month follow-up [25]. An upcoming Diabetes Body Project multisite trial across Norway, the Netherlands, and the US will compare the program to educational control through 2-year follow-up [30].

Due to the success of dissonance-based programming, several studies have focused on dissemination and implementation of the Body Project in the US, including comparing training models of varying intensity and exploring factors related to successful sustainability (e.g., positive attitudes towards the program, training new peer educators) [31–33]. Collectively, these studies highlight the adaptability and effectiveness of the Body Project as a front-line and scalable eating disorder prevention tool.

#### Mindfulness-Based Approaches

Mindfulness-based and embodied approaches to eating disorder prevention emphasize developing internal awareness (e.g., mindfully noticing hunger and satiety cues and fostering a sense of embodiment [the quality of a person’s lived experience in their body]), while embracing a non-diet, weight-inclusive approach. In recent years, these interventions have increasingly incorporated intuitive eating [34, 35], yoga [36–38], and self-compassion [39–44]. Notably, many of these programs are group-based, low-intensity, or virtually delivered, enhancing their accessibility and scalability. Intuitive eating interventions have ranged from a quasi-experimental universal school-based feasibility trial for early adolescents in New Zealand [35] to a mixed-methods evaluation of an uncontrolled pilot of a selective prevention program for U.S. college women with disordered eating [34]. These programs have demonstrated strong feasibility, as assessed through quantitative (scores > 4/5 [35]) and qualitative feedback from leaders [34], and acceptability (ratings > 2.79/3 [34] and > 70% agree/strongly agree the program was enjoyable [35]).

Yoga-based approaches have centered on the Eat Breathe Thrive (EBT) program, a yoga- and mindfulness-based intervention implemented primarily as a universal prevention model in the United States and United Kingdom [36, 37], with one selective application for Division I athletes [38]. EBT has been delivered both in person [36, 38] and virtually [37]. RCT results for the universal implementation have demonstrated small-to-medium improvements in eating disorder symptoms (η²*p* = .03), and small-to-large improvements in depression (η²*p* = .11), emotion dysregulation (η²*p* = .07), interoceptive awareness (η²*p* = .18), mindful self-care/eating (η²*p* = .03-0.06.06), and mindful eating (η²*p* = .03) post-intervention, compared to waitlist control, with some effects maintained at 6-month follow-up [36, 38]. EBT for athletes resulted in significant, medium improvements in body trust (η²*p* = .11) and mindful self-physical care (η²*p* = .12), but not eating disorder symptoms, compared to assessment-only control [36, 38].

Self-compassion-based interventions have also gained traction in the last several years, with applications ranging from universal delivery [42–44] to selective delivery for women with breast cancer [41] and women with internalized weight bias and higher weight [39, 40]. These studies were conducted in the United States [39, 40, 44], Canada [45], Australia [43], and China [41]. Several of the self-compassion-focused interventions have used micro-intervention formats (e.g., 3-minute writing exercises) [42–44] aimed at buffering against the impacts of social media [45] or negative eating experiences [43]. These programs have demonstrated significant small-to-moderate improvements on appearance dissatisfaction [45] and state body dissatisfaction [44] compared to attention control conditions. Haley and colleagues [39, 40] evaluated a 3-session self-compassion intervention to target internalized weight bias in women with higher weight in a case series [39] and pilot RCT compared to waitlist control [40]. Results from the pilot RCT suggested that the intervention resulted in greater improvements compared to waitlist control, with medium-to-large effects on internalized weight bias (η²*p* = .13,0.02), body shame (η²*p* = .30,0.15), and uncontrolled eating (η²*p* = .19,0.51) at post-intervention and 1-month follow-up, respectively. Collectively, these studies highlight the recent interest in and potential promise of mindfulness-based prevention strategies.

#### **Interventions Targeting Weight Stigma**

 Interventions Targeting Weight Stigma. Weight stigma encompasses the psychological and behavioral manifestations of weight bias, referring to negative attitudes, stereotypes, and discriminatory behaviors towards individuals in larger bodies. Current literature suggests that internalized weight bias, the process by which individuals adopt societal anti-fat attitudes and beliefs, is a significant modifiable risk factor for disordered eating, particularly among those with higher weight [46–49]. In the last several years, group-based interventions have focused on reducing weight stigma and internalized weight bias, selectively, for people in higher weight bodies [46, 49] and universally for individuals regardless of weight status [47, 48, 50]. These programs have used a variety of approaches and techniques including cognitive-behavioral [49], compassion or empathy-focused [46, 50], and dissonance-based [47, 48].

Research points to the effectiveness of group-based cognitive-behavioral counseling interventions for weight-bias internalization. This approach trains individuals to identify automatic negative thoughts related to their weight, and potential connections to feelings about higher-weight individuals. In an RCT among higher-weight youth in Thailand, participants in the intervention group reported significant and large improvements in weight-based internalization compared to counseling-as-usual (*d* = 1.60; calculated from means) [49]. Results suggest that cognitive-behavioral counseling may help reduce internalized weight stigma.

Compassion-focused interventions promote protective factors like self-kindness, emotion regulation, and empathy to address weight bias and anti-fat attitudes. A recent single-session intervention used fictional narratives to build greater empathy towards individuals in higher weight bodies and combat stereotypes around weight controllability [50]. Participant results from this RCT demonstrated significant improvements in overall weight bias and weight controllability compared to those in an informational control, with small-to-moderate effects at 1-month follow-up posttreatment (*η*_*p*_^2^ = 0.03–0.08) [50]. In an open trial using compassion-focused therapy groups for people in higher-weight bodies, Carter and colleagues [46] found significant and large reductions in body weight shame and external shame at three-month follow-up (*d* = 0.85; *d* = 1.01). These results highlight the initial promise of addressing external and internalized weight stigma with compassion-focused strategies.

Two programs have used dissonance-based, peer-led groups to target internal and societal impacts of anti-fat bias [47, 48]. In one RCT [48], Body Project participants endorsed significantly less thin-ideal internalization (*d* = 0.18), eating disorder symptoms (*d* = 0.16), and body dissatisfaction (*d* = 0.29) than waitlist control at post-intervention, although effects were small. These articles highlight the potential of cognitive behavioral and peer-led dissonance-based programming as an emerging area of research, with growing evidence for accessible group interventions addressing both internalized and systemic weight bias. However, further research is needed before these approaches can be established as first-line interventions.

### Specific Populations

#### **Programs Targeting Youth/School-Based Programming**

Over the past several years, various eating disorder prevention programs have focused on youth, with the majority adopting a universal prevention approach in school settings [51–55]. Several of these school-based interventions are classroom-based and include interactive elements, such as role-playing, group discussions, and homework. For example, Gordon and colleagues [56] conducted an RCT of the SoMe social media literacy program for Australian adolescents and observed significant decreases in dietary restraint at 6-month follow-up for girls (*d* = 0.24), and improved self-esteem for boys at the 6-month follow-up (*d* = 0.29) compared to a class-as-usual. In addition to school-based programs, multiple prevention programs have been developed to engage parental figures in reducing youth eating disorder risk. For example, Confident Body, Confident Child (CBCC) offers parents interactive, classroom-style sessions aimed at increasing knowledge of healthy lifestyle habits, self-esteem, and body confidence [57]. Results from an uncontrolled pilot demonstrated that CBCC produced significant small-to-moderate improvements in parental comprehension regarding body satisfaction and health habits (*d* = 0.46) [57].

Some programs have taken a selective approach to prevent eating disorders in at-risk populations. For example, Jones and colleagues [58] developed PaRent InterventiOn to pRevent dIsordered eating in children with TYpe 1 diabetes (PRIORITY), a psychoeducation program to prevent eating disorders in youth with Type 1 Diabetes. Results from an RCT found that PRIORITY resulted in significant small improvements in parent-reported child diabetes eating behavior (*d* = 0.10) and moderate improvements in child-reported diabetes eating problems (*d* = 0.50) at 3-month follow-up compared to waitlist. Another program, Goodform, aims to reduce muscle-enhancing supplement use and positive expectations surrounding anabolic androgenic steroid use among adolescent boys [59]. However, results from an RCT did not observe any significant improvements in muscularity concerns or attitudes towards steroid use compared to a treatment-as-usual at 8-week follow-up. Together, these diversified approaches highlight the need for both broad and targeted approaches in eating disorder prevention efforts in youth populations.

#### Programs Targeting Athletes or Gym-Goers

Several studies over the last four years have investigated eating disorder prevention interventions for athletes, given documented risk in this group. Due to the focused sampling, these studies have primarily been selective in nature [60–63], with a few indicated programs focusing on athletes with mild eating disorder symptoms [64, 65] and exercisers with symptoms of muscle dysmorphia [66]. The majority of this research in the last several years has included variants of the Female Athlete Body Project (FABP) [67, 68], a front-line intervention, which has recently been evaluated in elite female ballet dancers in the US [61], young ballet dancers of all genders in the US and Australia [60], younger athletes of all genders in Norway [69] and college-aged male athletes in the US [63], with some initial success in improving eating disorder risk factors. Unlike other Body Project variants, these programs do not use dissonance-based techniques; rather, they focus on defining healthy body ideals, increasing nutritional density, addressing relative energy deficiency, and factors that might influence performance outside of weight (sleep, time off, etc.) in 3 peer-led sessions. These FABP adaptations have largely kept the FABP structure, but have modified language to be suitable/applicable to different populations (e.g., ballet dancers, boys/men).

Outside of the FABP, MOPED-A: Motivational and Psycho-Educational Self-Help Programme for Athletes with Mild Eating Disorder Symptoms, an indicated prevention program has resulted in strong initial efficacy in an open trial among 35 athletes with disordered eating (77.1% women), including large reductions in eating disorder symptoms post-intervention and 1-month follow-up (r = .45-.55) and over one-third of participants seeking further help for their eating post-treatment [70]. These studies highlight promising strides toward addressing eating disorder risk in athletic populations, largely in the US and in Norway. Additional research is needed to assess the long-term efficacy of these interventions across diverse athletic communities across the globe.

## Digital-Based Interventions

With the increasing use of technology across the globe, numerous eating disorder prevention programs have begun to incorporate innovative technological components. These digital-based programs range from app-based self-help programs [71–73], to online-based therapy modalities [74–77], to chatbots [78–80], and single-session interventions [80–82]. Self-help innovations, such as the mHealth app have demonstrated favorable outcomes in a RCT, reducing body dissatisfaction and increasing self-compassion and body appreciation, with significant small to large effects for girls (*η*_*p*_^2^ = 0.027–0.27) and significant small effects for boys (*η*_*p*_^2^ = 0.007–0.024) compared to waitlist control at 4-week follow-up [73]. Internet-based therapy programs have also been evaluated. For example, an RCT of Internet-Cognitive Behavioral Therapy for Perfectionism {O’Brien, 2022 #6611} resulted in a greater number of people achieving reliable change in perfectionism (57.14% vs. 0%) and eating disorder symptomatology (71.43% vs. 14.29%) compared to waitlist at 4-week follow-up. Recent research has also explored chatbot-based preventative programs. Results from an open trial suggest that participant chatbot use yielded significant moderate increases in body satisfaction (*d* = 0.54) [80], while a RCT showed that participant chatbot use produced significant but small decreases in weight/shape concerns (*d* = 0.19) compared to waitlist control at 3-month follow-up [79]. Chan and colleagues [78] also underscored key challenges of chatbot-delivered interventions, such as their limited ability to respond to complex user concerns that fall outside of predefined scripts, strict character limits that constrain communication, and the absence of emotionally nuanced responses that typically require human moderation. These findings emphasize the ongoing discourse surrounding the advantages and disadvantages of closed-loop versus generative chatbot systems.

Another recent digital-based innovation in eating disorder prevention is the use of single-session interventions targeting core mechanisms linked to eating disorder onset/maintenance [83]. These interventions have demonstrated significant moderate-large improvements in functional appreciation (*d* = 0.72) and body dissatisfaction (*d* = 0.61) in an open trial for adolescents with body image and mood problems [82] and significant, large effects for body image concerns (*d* = 0.74) in individuals with binge eating compared to waitlist control at 8-week follow-up [81]. Notably, the majority of recent digital-based programs are selective or indicated, focusing on offering resources to those who display elevated risk for an eating disorder or early symptomatology. Given the novelty of this area of research, at this time, there are no specific digital-based programs that would be categorized as first-line interventions. As technology continues to evolve, these brief, digital-based programs represent a promising and accessible avenue for scaling eating disorder prevention efforts.

## System Level Interventions

While many existing programs target eating disorder prevention at an individualized level, addressing structural factors that contribute to or sustain risk are vital to creating meaningful, population-level impact. At the systems level, eating disorder prevention efforts in the last several years have ranged from public policy initiatives [84, 85] to programming in primary care [86] and community health settings [87, 88]. Articles like Long [85], a cost-effectiveness analysis modeling, and Austin [84], a case study and policy evaluation, conceptualize eating disorders as public health issues, advocating for population-level, cost-effective prevention strategies created with stakeholder involvement. This framing shifts away from individualized, reactive care toward preventative, systemic approaches. Among multiple studies, there has been a shared push to equip frontline health workers with developmentally appropriate tools to identify eating disorder risk and intervene early. Indeed, several papers [86, 87, 89] emphasize embedding eating disorder prevention and early intervention strategies into routine care provided by primary care providers and community health nurses. In Australia, the Confident Body, Confident Child [87] program, engages parents of children aged 2–6, promoting skills related to positive food-talk and body satisfaction. The Mealtime Chatter Matters program, designed for implementation within primary care centers, encourages mothers of newborns to attend preventative sessions with community health nurses, who provide behavioral strategies for positive framing and dialogue around food [89]. Family-focused programs offer a unique lens into improving outcomes through parent psychoeducation, shared motivation for change, and reduced inpatient needs. Using focus groups, Norton and colleagues [88] built off of past programs and co-designed the program with healthcare practitioners and community members, suggesting a movement toward collaborative, context-sensitive solutions, rather than top-down implementation. Front-line research on system-level interventions settles into two branches, policy change [84, 85] and implementation [87, 89]. Primary and community care prevention efforts, such as the Mealtime Chatter Matters program and the Confident Body Confident Child program, reflect a growing recognition that eating disorder prevention requires integrative and community-embedded strategies that prioritize early detection, primary care involvement, and culturally responsive design at scale.

## Early Intervention Efforts

Although many of the above efforts have focused on eating disorder prevention in non-clinical populations, early intervention programs target individuals newly diagnosed or in early stages of the illness to reduce symptomology and improve quality of life. Recent research on early intervention programs has demonstrated efficacy in reducing eating disorder symptom severity, shortening illness duration, and decreasing service utilization when compared to treatment-as-usual. Much of this work is rooted in Australia and the United Kingdom (UK), where early intervention programs have been built into the robust national healthcare services. One such initiative, First Episode Rapid Early Intervention for Eating Disorders (FREED), was developed in the UK to support the National Health Service in facilitating more rapid access to high-quality care [90]. With the implementation of FREED, patients experienced a significantly reduced duration of untreated illness (4 months shorter than treatment-as-usual) [90, 91]. Over half of FREED participants were weight restored after 12 months, compared with 17.9% of treatment-as-usual participants [90–92]. Qualitative feedback from clinicians and administrators indicated general support for the FREED model [93, 94]. Challenges related to illness severity and systemic organizational barriers, such as limited resources, competing clinician demands, and lack of knowledge of the program in referral settings, were identified as impediments to wider implementation [93, 94]. In response to the global complexities surrounding access to prevention and early intervention efforts in the UK, Hemmings and colleagues [95] proposed the Eating Disorders: Improving Experience (EDIFY) research project, a four-year interdisciplinary research program incorporating six interrelated workstreams aimed at examining the recovery processes through a biopsychosocial lens.

Early intervention efforts can also be scaled across regional services and adapted into public health models [88, 94]. One such example is emerge-ED, an early intervention model evaluated for use at primary health care centers [96]. Modelled after FREED, this program aims to bring eating disorder treatment services to low socio-economic status areas in Australia. Findings indicated positive replication of its effectiveness at reducing eating disorder symptoms at 70 days since the start of treatment, with medium-to-large improvements in eating disorder cognitions (*d* = 0.63) and dietary restriction (*d* = 0.70), and small improvements in objective binges (*d* = 0.17) and driven exercise (*d* = 0.23) [96]. Implementing in a primary care setting also addressed common barriers associated with treatment, such as denial of illness severity and long waitlists.

Beyond primary care interventions, families and caregivers can also play a critical role in fostering recovery-oriented environments. Rosello and colleagues [97] published an open trial designed to estimate the impact of a six-week group intervention, conducted with parents of newly diagnosed children with anorexia and atypical anorexia. The intervention, offered at the point of referral to eating disorder services, was associated with weight gain (*η*_*p*_^2^ = 0.44), a predictor of treatment success, and large improvements in eating disorder symptomology (*η*_*p*_^2^ = 0.43), with effects maintained at the 6-month follow-up [97]. Early interventions addressing eating disorders at both structural and interpersonal levels may be successful in providing comprehensive, early-stage support, with public health initiatives like FREED identified as the current first-line approach.

## Conclusions

Over the past four years, eating disorder prevention research has increasingly focused on developing and adapting personalized interventions for specific populations across varied global contexts. Concurrently, there has been growing emphasis on addressing internalized weight bias and fat phobia, aligning with shifts toward promoting body neutrality over prescriptive body positivity. Future efforts should continue to prioritize expanding these interventions to underserved populations (e.g., racially and ethnically marginalized groups, those experiencing food insecurity), particularly in non-Western contexts. Further, as many interventions integrate multiple components (e.g., intuitive eating, self-compassion, dissonance, weight stigma reduction), future studies should employ dismantling and comparative effectiveness designs to isolate active ingredients.

In response to persistent barriers to eating disorder intervention access and affordability, recent prevention efforts have emphasized scalability and accessibility, particularly through group-based formats and digital technologies. The rise of digital interventions—including mobile apps, chatbots, and single-session interventions—has accelerated since the COVID-19 pandemic, with growing evidence supporting their feasibility and acceptability. Continued investigation into the utility of digital (and non-digital) micro-interventions and single-session approaches targeting specific mechanisms is also warranted. Notably, advances in digital interventions are not meant to replace in-person prevention programs, but rather can be used as a helpful tool to work in tandem with these efforts. Critically, future digital research should continue to explore the responsible and ethical use of AI, particularly generative AI in delivering personalized and appropriate care.

Further, there is increasing recognition that effective eating disorder prevention must be embedded within broader systems, including family environments, schools, primary care, public policy, and community health. Much of this systems-level work has emerged from countries with centralized healthcare infrastructure; adaptation and implementation in less centralized contexts remain an important next step. Relatedly, integrating caregivers into prevention programs, while being attentive to caregiver burden, and addressing additional ecological factors in children’s lives will be essential. Training frontline health professionals with appropriate tools for early detection and intervention remains a critical priority.

Finally, dissonance-based programs, which have demonstrated strong efficacy, should continue to be adapted for diverse populations and implemented across varied settings(e.g., community clinics, primary care). However, most other modalities lack long-term follow-up data. Assessing the durability of intervention effects and their role in preventing full-threshold eating disorder onset will require sustained funding and global advocacy for future prevention research.

## Data Availability

No datasets were generated or analysed during the current study.
